# Impact of high hemoglobin levels on carotid artery intima–media thickness and its predictive value for hypertension in high-altitude areas: a real-world study

**DOI:** 10.3389/fcvm.2024.1429112

**Published:** 2025-01-09

**Authors:** Long Hongyu, Long Ga, Zhang Yiqian, Xu Qiuyu, Li Kemin, Qing Maiyongcuo, Xiong Min

**Affiliations:** ^1^ Hyperbaric Oxygen, Chengdu First People’s Hospital, Chengdu, Sichuan, China; ^2^ Internal Medicine Department, Dege County People’s Hospital of Ganzi Prefecture, GanZi, Sichuan, China; ^3^ Internal Medicine Department, The Second People’s Hospital of Jiangyang District, Luzhou City, Sichuan, China; ^4^ The Department of Obstetrics and Gynecology, West China Second University Hospital of Sichuan University, Chengdu, Sichuan, China

**Keywords:** carotid intima-media thickness, hemoglobin, hypertension, plateau region, predictive value, real-world study

## Abstract

**Background and purpose:**

The hemoglobin levels in the peripheral blood of individuals living at high altitudes are significantly higher than normal levels. These levels are closely associated with atherosclerosis and cardiovascular events. This study aimed to investigate the correlation between hemoglobin levels in the peripheral blood and hypertension in high-altitude regions, providing a basis for preventing and treating primary hypertension in these regions.

**Materials and methods:**

From May 2020 to May 2021, patients diagnosed with primary hypertension in plateau regions of China were selected as participants. The clinical data, including lifestyle habits and blood biochemical indicators, were collected from the clinical case database for patients meeting the inclusion criteria. The logistic regression analysis was performed to identify factors influencing carotid intima–media thickness in patients with primary hypertension in plateau regions. The ROC curve was plotted to analyze the impact of peripheral blood hemoglobin levels on hypertension, determine the hemoglobin threshold for predicting hypertension in plateau areas, and evaluate the predictive value of hemoglobin level for hypertension.

**Results:**

A total of 200 patients (105 men with an average age of 64.8 ± 12.75 years and 95 women with an average age of 69.5 ± 11.54 years) were enrolled in this study. Logistic regression analysis revealed that age, CO_2_-CP, ALT, APOB, CRP, and HGB were independent risk factors for increased carotid artery intima–media thickness (*P* < 0.05). The hemoglobin threshold for predicting hypertension in high-altitude areas was 131 g/L. The area under the ROC curve for predicting hypertension with elevated hemoglobin level was 0.799 (0.719–0.880).

**Conclusion:**

Elevated hemoglobin levels contribute to the thickening of the carotid artery intima–media layer and hold predictive value for high-altitude hypertension.

## Highlights

• Hemoglobin levels effect carotid artery intima–media thickness
• High hemoglobin predictive value for hypertension in plateau regions

## 1 Introduction

Hypertension is one of the most common cardiovascular diseases globally, posing a significant risk of conditions such as stroke, coronary heart disease, and heart failure, leading to mortality among humans. According to the largest hypertension research report globally, the number of adults with hypertension, aged 30–79 years, increased from 650 million to 1.28 billion between 1990 and 2019 (1).

The primary pathological feature of hypertension is arteriosclerosis, which affects both large and small arteries. The thickness of the carotid artery intima-media (CIMT) serves as a marker for vascular structural abnormalities, such as atherosclerosis, and is of significant importance for the prevention and treatment of hypertension. Hypertension acts as a catalyst for the thickening of the carotid intima-media. Prolonged hypertension can alter hemodynamics, damage endothelial cells, and mediate the adhesion of lipids and platelets to the vascular wall, gradually resulting in increased CIMT. Hypertension and CIMT thickening influence each other; sustained high blood pressure accelerates the thickening process, increasing the risk of cardiovascular and cerebrovascular diseases. Conversely, increased CIMT can elevate blood pressure, creating a vicious cycle. Studies have shown a close correlation between IMT thickening and the onset and progression of hypertension, reflecting pathological changes in the vascular wall and its response to hemodynamic factors. By monitoring carotid IMT, cardiovascular risks in hypertensive patients can be identified early, guiding clinical interventions that help reduce the incidence of cardiovascular events (2–4). Therefore, carotid IMT plays an increasingly important role in the early diagnosis and management of hypertension, and exploring the factors influencing intima-media thickening is crucial for the prevention and treatment of hypertension.

The global plateau region is vast, with a permanent population of 140 million (2). The unique environmental factors of the plateau region may affect the occurrence and development of hypertension. Studies indicate that the prevalence of hypertension is significantly higher in plateau regions than in plain areas. Moreover, the incidence of hypertension also significantly increases with the increase in altitude (5, 6). Therefore, investigating the risk factors associated with primary hypertension in plateau regions is essential for its prevention, diagnosis, and treatment.

Previous studies have indicated a correlation between hemoglobin levels and hypertension, with an increase in hemoglobin typically thought to promote elevated blood pressure through its effects on blood viscosity and hemodynamics (7–10). In high-altitude regions, where oxygen levels are lower, individuals often experience a significant increase in hemoglobin to adapt to the hypoxic environment. While this adaptation enhances oxygen transport, it may also raise the risk of hypertension. However, the relationship between elevated hemoglobin levels and increased blood pressure in high-altitude populations has not been thoroughly investigated. Some studies have observed lower blood pressure levels among residents of high-altitude areas; however, the underlying mechanisms may involve complex environmental factors, genetic backgrounds, and individual physiological characteristics. Additionally, previous research often lacked large sample sizes and longitudinal data, failing to adequately consider the impact of lifestyle and other comorbidities on hypertension. Therefore, further exploration of the relationship between hemoglobin levels and blood pressure in high-altitude populations is crucial for providing a more comprehensive understanding and clinical guidance, highlighting its research significance.

This study examined the predictive significance of different hemoglobin levels for hypertension among populations residing in high-altitude regions, using CIMT as an early indicator of arterial sclerosis. The study further explored the impact of hemoglobin levels on hypertension in this population, offering insights for the development of hypertension prevention and management strategies tailored to high-altitude areas.

## 2 Materials and methods

### 2.1 Research objectives

This retrospective study, conducted in real-world settings, enrolled patients diagnosed with primary hypertension in plateau regions from May 2020 to May 2021. All patients came from Dege County People's Hospital of Ganzi Prefecture, GanZi, Sichuan, China. The diagnostic criteria for hypertension included blood pressure measurements taken three times on different days without antihypertensive medication, with systolic blood pressure ≥140 mmHg and/or diastolic blood pressure ≥90 mmHg. The diagnosis of carotid atherosclerosis relied on carotid ultrasound examination, with thickening of the intima–media layer defined as a maximum intima–media thickness (IMT) of the common carotid artery ≥1.0 mm, or maximum IMT of the carotid sinus ≥1.2 mm, and carotid plaque defined as IMT ≥1.5 mm (7). Patients with malignant hypertension, secondary hypertension, and those with conditions such as diabetes affecting CIMT were excluded from the study. Patients with diseases that affect hemoglobin levels, such as hematological malignancies and unexplained anemia, were also excluded. This study received ethical approval from Chengdu Hospital of Integrated Traditional Chinese and Western Medicine and was exempted from informed consent.

### 2.2 Data collection

Patient data were retrospectively collected from the clinical medical records database, encompassing information such as age, sex, ethnicity, body mass index (BMI), waist circumference, lifestyle habits (diet and sleep), and underlying diseases, as well as blood routine, full blood lipids (fasting venous blood), fasting blood sugar, uric acid, myocardial enzymes, procalcitonin, and other blood biochemical indicators. Additionally, routine urine tests (morning midstream urine tests) were included in the dataset.

### 2.3 CIMT measurement

Ultrasound examinations were conducted using a color Doppler ultrasound detector (American GEe9, equipped with a 10-MHz linear array probe). During the procedure, the patient lay supine with their head slightly turned to the opposite side. Longitudinal and transverse scans were performed along the carotid artery, covering both sides, the carotid artery bifurcation, and the internal and external carotid arteries from various planes and angles. The CIMT and plaque status in the blood vessels were observed. In the ultrasound images, the carotid artery vessel wall appeared as two slightly bright parallel linear echoes, with the outer line representing the vessel's outer membrane and the inner line representing the vessel's intima. The vertical distance between these two lines indicated the thickness of the carotid artery intima. Measurements of the carotid artery intima–media thickness were obtained at three specific locations: the far end near the bifurcation of both carotid arteries, 1 cm above the bifurcation, and 1 cm above the start of the internal carotid artery on the back wall. The average of these measurements was calculated to determine the CIMT.

### 2.4 Statistical analysis

Statistical analysis was conducted using SAS 9.4 statistical software. Descriptive statistical analysis was initially performed on various indicators. The Shapiro-Wilk test was used to test for normality of distribution, and the Levene's test was used to test for the stability of variance. Quantitative data that conforms to a normal distribution were presented as mean (standard deviation) to indicate central tendency and dispersion, whereas qualitative data were expressed as rates or percentages. Logistic regression analysis was used to identify factors influencing intima–media thickness of the carotid artery. A *P* value <0.05 indicated a statistically significant difference. Furthermore, an ROC curve was plotted to predict hypertension using hemoglobin levels. The cutoff value for predicting hypertension and the area under the ROC curve for hemoglobin levels were calculated. We will use the Youden index to select the cutoff value. The Youden index is a statistical measure used to evaluate the effectiveness of diagnostic tests. It is particularly useful for determining the optimal threshold that distinguishes between two groups, typically those with and without a specific disease. The index is defined as the maximum vertical distance between the receiver operating characteristic (ROC) curve and the no-discrimination diagonal, which represents random guessing. This index helps assess the balance between the sensitivity and specificity of the test.

## 3 Results

### 3.1 Basic characteristics of the participants

The study enrolled 200 patients, including 105 men with an average age of 64.8 ± 12.8 years and 95 women with an average age of 69.5 ± 11.5 years. The majority of the study participants were Tibetan (99.5%), with farmers and herders constituting 86% of the occupations. More than 90% of the patients reported no history of smoking or alcohol consumption. Additionally, 31% of the patients were classified as obese. The basic characteristics of the study participants are summarized in Tables 1, 2.

**Table 1:** Basic characteristics of participants (part 1).

Variables	*n* (%)/Mean ± STD
Gender	
Male	105 (52.5%)
Female	95 (47.5%)
Age	67.0 ± 12.4
Weight	70.4 ± 13.7
Height	164.5 ± 7.8
Nationality	
Tibetan	199 (99.5%)
Han nationality	1 (0.5%)
Profession	
Employees of enterprises and institutions	21 (10.5%)
Self-employed/employed	7 (3.5%)
Farmer	145 (72.5%)
Herdsmen	27 (13.5%)
Smoking history	
No	188 (94.0%)
Yes	12 (6.0%)
Drinking history	
No	189 (94.5%)
Yes	11 (5.5%)
Diet situation	
Mainly rice	23 (11.5%)
Mainly highland barley	168 (84.0%)
Ainly rice + highland barley	7 (3.5%)
Mainly rice + highland barley + pasta	2 (1.0%)
Love food	
Meat-based	7 (3.5%)
Meats, vegetables and fruits	4 (2.0%)
Meats, vegetables, fruits, eggs, soy products	188 (94.0%)
Vegetarian diet	1 (0.5%)
Sleep time per day	
<6 h	10 (5.0%)
6–8 h	103 (51.5%)
9–12 h	86 (43.0%)
>12 h	1 (0.5%)
BMI	
<18.5	5 (2.5%)
18.5–23.9	66 (33.0%)
24–27.9	67 (33.5%)
≥28	62 (31.0%)
Waistline	
Abdominal obesity in men	76 (72.4%)
Abdominal obesity in women	71 (74.7%)
Family history	
No	194 (97.0%)
Hypertension	2 (1.0%)
Diabetes	1 (0.5%)
Unknown	3 (1.5%)

**Table 2:** Basic characteristics of participants (part 2).

Variables	*n* (%)/Mean ± STD
Admission blood pressure	
Diastolic blood pressure	95.9 ± 18.6
Systolic blood pressure	149.6 ± 28.6
Hypertension	140 (70.0%)
Normal blood pressure	54 (27.0%)
Unknown	6 (3.0%)
CIMT	
Left	1.0 ± 0.3
Right	1.0 ± 0.2
Thicken	132 (66.0%)
Norman	68 (34.0%)
Hemoglobin	150.0 ± 31.9
Abnormal (−)	19 (9.5%)
Normal	68 (34.0%)
Abnormal (+)	88 (44.0%)
Unknown	25 (12.5%)
Carotid atherosclerotic plaque formation	
Yes	154 (77.0%)
No	46 (23.0%)
Number of hardened plaques	
<2↑	128 (64.0%)
≥2↑	72 (36.0%)

CIMT, carotid intima-media thickness; STD, standard deviation; +, indicates greater than normal threshold; −, indicates less than the normal threshold.

### 3.2 Factors influencing the CIMT in logistic regression analysis

Age, sex, BMI, smoking history, alcohol history, waist circumference, blood test results, and so forth were the probable influencing factors in the logistic regression analysis (Table 1). The results indicated that age, CO_2_-CP, ALT, APOB, CRP, and HGB were the probable influencing factors for the thickening of carotid intima–media (*P* < 0.05), with odds ratio (OR) and 95% confidence interval (CI) of 1.692 (1.059–2.704), 0.475 (0.290–0.777), 0.211 (0.091–0.489), 2.012 (1.131–3.579), 6.945 (1.177–40.972), and 0.540 (0.307–0.949), respectively. Conversely, the following factors raise the risk: CRP (up to seven times) and ApoB (it doubles). Further details of the logistic regression analysis are depicted in Table 3.

**Table 3:** Logistic regression analysis of influencing factors of carotid artery intima-media thickness.

Variables	DF	PE	STD ERR	Chi-square value	*P*	OR	OR 95%CI	
Gender	1	0.366	0.302	1.470	0.225	1.442	0.798	2.606
Age	1	0.526	0.239	4.839	0.028	1.692	1.059	2.704
Smoking history	1	−0.975	0.817	1.424	0.233	0.377	0.076	1.871
Drinking history	1	0.287	1.068	0.072	0.789	1.332	0.164	10.811
Diet situation	1	0.010	0.013	0.559	0.455	0.010	−0.016	0.036
Love food	1	−0.298	0.357	0.698	0.404	0.742	0.369	1.494
Weight	1	−0.006	0.011	0.264	0.608	0.994	0.973	1.016
Height	1	−0.009	0.019	0.207	0.649	0.991	0.955	1.029
BMI	1	−0.056	0.180	0.098	0.754	0.945	0.665	1.344
Waistline	1	0.246	0.339	0.526	0.468	1.279	0.658	2.486
ALP	1	0.687	0.445	2.356	0.125	1.979	0.828	4.730
LDH	1	−0.099	0.279	0.125	0.724	0.906	0.524	1.566
CO2-CP	1	−0.744	0.251	8.792	0.003	0.475	0.290	0.777
CREA	1	0.625	0.337	3.445	0.064	1.869	0.966	3.616
ALT	1	−1.554	0.428	13.175	0.000	0.211	0.091	0.489
AST	1	−0.782	0.537	2.118	0.146	0.457	0.160	1.312
γ-GT	1	−0.151	0.310	0.236	0.628	0.860	0.469	1.580
TG	1	0.009	0.412	0.001	0.983	1.009	0.450	2.264
TCHO	1	0.242	0.334	0.522	0.470	1.273	0.661	2.453
HDL-C	1	0.338	0.261	1.670	0.196	1.402	0.840	2.340
ApoA1	1	−0.205	0.288	0.507	0.477	0.814	0.463	1.433
ApoB	1	0.699	0.294	5.651	0.017	2.012	1.131	3.579
LDL-C	1	0.541	0.318	2.902	0.089	1.718	0.922	3.203
CRP	1	1.938	0.906	4.580	0.032	6.945	1.177	40.972
WBC	1	−0.056	0.390	0.021	0.886	0.946	0.441	2.029
RBC	1	−0.401	0.304	1.749	0.186	0.669	0.369	1.214
HGB	1	−0.617	0.288	4.595	0.032	0.540	0.307	0.949
PLT	1	−0.070	0.398	0.031	0.861	0.933	0.428	2.034
ESR	1	0.629	0.531	1.406	0.236	1.876	0.663	5.306

PE, parameter estimation; DF, degree of freedom.

### 3.3 Predictive value of peripheral blood hemoglobin for high-altitude hypertension

**Figure 1:**
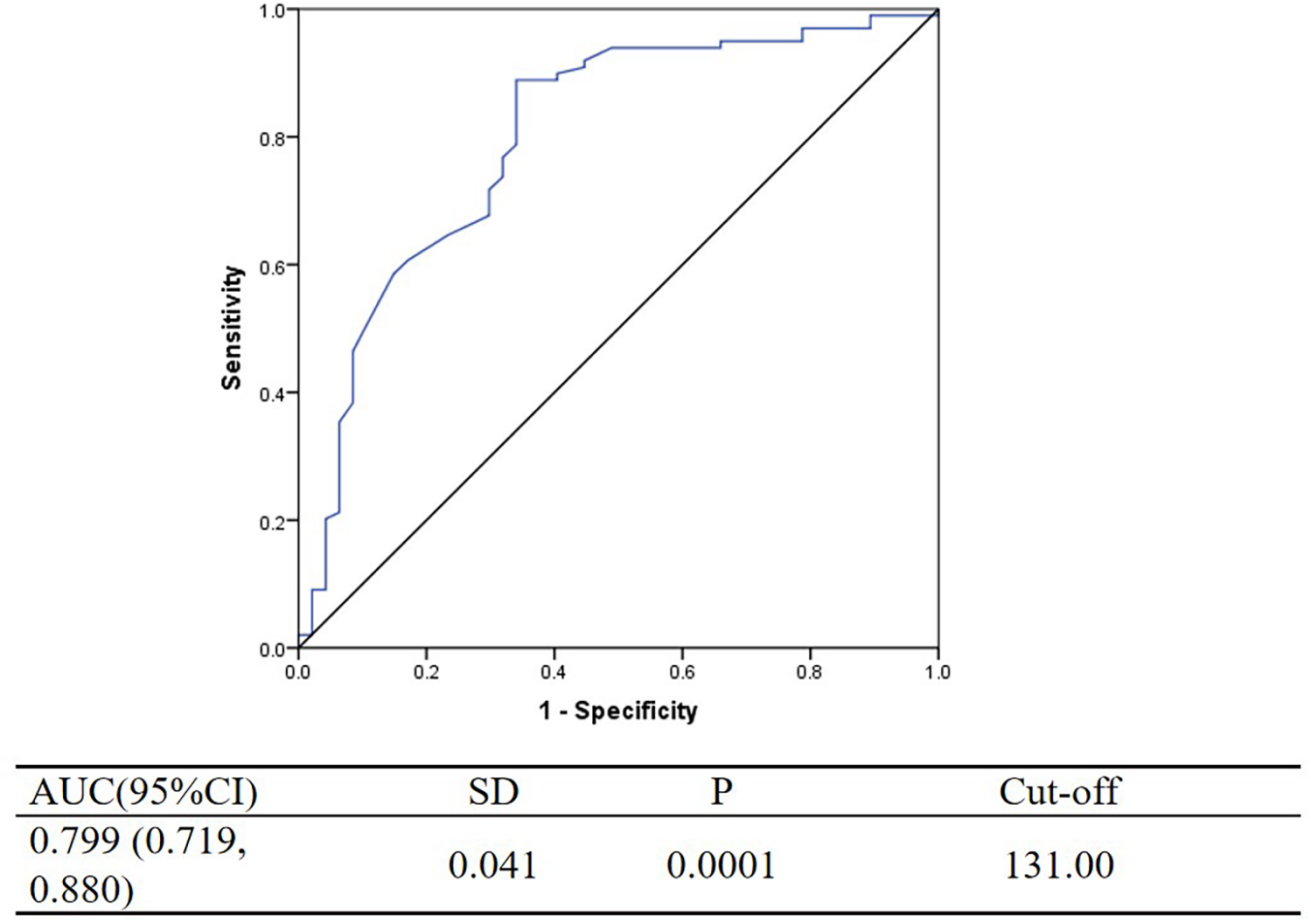
The ROC curve depicting the predictive ability of peripheral blood hemoglobin for high blood pressure in high-altitude areas. AUC, area under curve; ROC, receiver operating characteristic.

The ROC curve depicting the predictive ability of peripheral blood hemoglobin for high blood pressure in high-altitude areas is shown in Figure 1. The threshold for HGB predicting high blood pressure in high-altitude regions is determined to be 131 g/L. Based on this threshold, the study participants were categorized into high-hemoglobin and low-hemoglobin groups. When peripheral blood hemoglobin levels were ≥131 g/L, the area under the ROC curve for predicting high blood pressure in high-altitude regions was 0.799, with its 95% CI = 0.719–0.880.

## 4 Discussion

Several studies have indicated a correlation between elevated hemoglobin levels and hypertension (11, 12). Huang Jianyu (12) and others explored the association between peripheral hemoglobin and arteriosclerosis. Their study involved 419 randomly selected hypertensive patients, and multiple logistic regression analysis identified hemoglobin as an independent risk factor for severe (OR = 1.025, 95% CI: 1.003–1.045, *P* < 0.05) and moderate (OR = 1.035, 95% CI: 1.008–1.056, *P* < 0.05) arteriosclerosis in patients with hypertension. Furthermore, multifactor analysis in this study revealed a positive correlation between hemoglobin levels and CIMT, suggesting that higher hemoglobin levels increase the risk of hypertension development and hold the potential as a predictor of hypertension.

The mechanism by which increased hemoglobin levels increase the risk of hypertension remains unclear. Existing research suggests several potential mechanisms: (1) Activation of renin–angiotensin–aldosterone system (RAAS): Activation of RAAS leads to the conversion of renin into angiotensin II, which promotes vasoconstriction. Additionally, other tissues may produce angiotensin II and stimulate the production of erythropoietin. (2) Damage of endothelial cells: Endothelial cell damage may affect both blood pressure regulation and hemoglobin concentration. (3) Increased levels of growth factor: Elevated growth factor levels may promote endothelial cell damage, and these levels are positively correlated with hemoglobin levels. Additionally, studies have shown a positive correlation between serum hepatocyte growth factor levels and hypertension, potentially increasing the risk of hypertension.

In high-altitude regions, environmental changes such as increased altitude lead to hypoxia, resulting in elevated hemoglobin levels in the population. High hemoglobin levels not only contribute to atherosclerosis but also promote hypertension (8). Studies have indicated that elevated blood hemoglobin levels may increase the risk of hypertension in high-altitude populations (8). This study analyzed the risk factors for the thickening of carotid artery intima–media in patients with hypertension using multifactorial logistics regression analysis. The findings revealed that hemoglobin was a high-risk factor for carotid artery intima–media thickening in patients with hypertension, with a threshold of 131 g/L, which holds promise for predicting high-altitude hypertension. Simultaneously measuring hemoglobin levels and blood pressure can effectively predict the occurrence of hypertension. Studies have shown that elevated hemoglobin levels are often associated with an increased risk of hypertension. By integrating these two important indicators, it becomes easier to identify potential hypertensive patients, thereby providing a basis for early intervention. Regular monitoring of hemoglobin and blood pressure not only aids in assessing cardiovascular health but also guides personalized treatment in clinical practice, enhancing the effectiveness of hypertension management. This underscored the potential value of peripheral blood hemoglobin detection in the early identification and prevention of hypertension in high-altitude populations.

The average hemoglobin (HGB) level among hypertensive individuals in China typically ranges from 120 to 140 g/L, with men generally exhibiting higher HGB levels than women. Studies indicate that in high-altitude areas, the HGB levels of residents are significantly higher than those of individuals living in flatland regions due to the effects of a hypoxic environment. Elevated HGB levels reflect the body's ability to adapt to low oxygen conditions, making it particularly important to maintain appropriate HGB levels in these areas. Normal HGB levels not only facilitate effective oxygen transport but also reduce the risk of carotid intima-media thickening, consequently decreasing the incidence of hypertension and its associated cardiovascular diseases. In high-altitude regions, sustaining optimal HGB levels is crucial for lowering the risk of carotid intima-media thickening, thereby improving overall cardiovascular health. Therefore, enhancing the monitoring and intervention of HGB levels in hypertensive populations will provide a solid foundation for preventing cardiovascular events.

In conclusion, this real-world study investigated the correlation between elevated peripheral blood hemoglobin levels and hypertension, as well as CIMT, in high-altitude populations. High hemoglobin levels can predict hypertension in these populations. However, given the limitations of this being a single-center, small-sample study, further validation from large-sample, high-quality studies is warranted.

## Data Availability

The original contributions presented in the study are included in the article/Supplementary Material, further inquiries can be directed to the corresponding author.
